# A virtual reality paradigm as an analogue to real-life trauma: its effectiveness compared with the trauma film paradigm

**DOI:** 10.1080/20008198.2017.1338106

**Published:** 2017-06-14

**Authors:** Anne A. Cuperus, Fayette Klaassen, Muriel A. Hagenaars, Iris M. Engelhard

**Affiliations:** ^a^ Health, Medical and Neuropsychology, Leiden University, Leiden, The Netherlands; ^b^ Triple, Alkmaar, The Netherlands; ^c^ Clinical Psychology, Utrecht University, Utrecht, The Netherlands

**Keywords:** PTSD, intrusions, trauma film paradigm, virtual reality

## Abstract

**Background**: The trauma film paradigm (TFP) is a well-established method to study the effects of analogue psychological trauma under controlled laboratory settings. It has been used to examine pre-, peri-, and post-trauma processes, and to create and test interventions. A possible drawback is that watching films is a somewhat passive endeavour that lacks active behavioural engagement. Virtual reality (VR) may provide a better alternative. Like the TFP, VR allows for experimental control. In addition, it can induce a greater ‘feeling of presence’ and allows interaction with the environment, enabling research on action–reaction associations.

**Objective**: We aimed to validate the utility of a VR paradigm as an experimental model to study psychological trauma by comparing its effectiveness with the TFP.

**Method**: One group of participants (*N* = 25) was shown an aversive film, and another group (*N* = 25) moved through a VR scene. Main outcome measures were intrusion frequency assessed with a 7-day diary and self-rated vividness and emotionality of recalled memories related to the film or VR scene.

**Results**: The results indicate that the film and VR scene were equally effective in inducing vivid and intrusive memories. However, self-reported emotional intensity appeared to be higher for memories related to the film than for memories related to the VR scene.

**Conclusions**: Perhaps the film was more effective in inducing emotional memories than the VR scene due to its more aversive content. However, the VR scene seemed equally effective in inducing vivid and intrusive memories, and merits further exploration in light of ethical considerations (less aversive content) and other presumably beneficial qualities (e.g. inducing a greater feeling of presence and allowing interaction with the environment).

Post-traumatic stress disorder (PTSD) is a mental disorder that can develop after a person is exposed to a traumatic event. In the 5th Edition of the Diagnostic and Statistical Manual of Mental Disorders (DSM-5; American Psychiatric Association, 2013), a traumatic event is described as exposure to actual or threatened death, serious injury, or sexual violence. In case of PTSD, this exposure leads to persistent re-experiencing of the traumatic event (e.g. intrusive memories, which are considered to be the hallmark symptom of PTSD; James et al., 2016), persistent avoidance of stimuli associated with the trauma, hyperarousal, and negative alterations in cognitions and mood. In a sample of nearly 3000 American adults, about 89.7% reported exposure to at least one traumatic event in their lifetime, and 8.3% had developed PTSD (cf. DSM-5 criteria; Kilpatrick et al., 2013).

A better understanding of the basic mechanisms underlying trauma symptom development is important because it provides insight into how symptoms can be reduced. Clinical studies may be useful in this respect, but a limitation of such studies is that they often rely on retrospective reports of trauma-related reactions many years later. As argued by Candel and Merckelbach (2004), this is problematic because people in general, and patients with PTSD in particular, find it difficult to give accurate descriptions of past emotional states. Moreover, reports of memory for traumatic events often change over time (Engelhard, van den Hout, & McNally, 2008) because individuals may interpret memories differently over time (Engelhard & McNally, 2015; see also Lommen, van de Schoot, & Engelhard, 2014). Experimental analogues are therefore warranted (James et al., 2016). The trauma film paradigm (TFP) is a well-established alternative method which involves showing non-clinical participants unpleasant films. Unpleasant film viewing as an experimental paradigm was introduced by Lazarus (1964), and was then further refined to study factors related to the development of intrusive thoughts (Horowitz, 1969) and intrusive images (Holmes, Brewin, & Hennessy, 2004) related to the film. The TFP is useful because it offers experimental control and the trauma films typically depict the types of events listed as traumatic in the DSM-5 (events involving actual or threatened death, serious injury, or sexual violence). Moreover, exposure to trauma films elicits measurable responses analogous to symptoms experienced during and shortly after viewing a traumatic event in real life (James et al., 2016), such as increases in negative mood (Clark, Mackay, & Holmes, 2015), and intrusive memories of the film (Holmes & Bourne, 2008; James et al., 2016). The TFP has been used to test pre-, peri-, and post-trauma processes; e.g. mechanisms of memory formation, and vulnerability factors. It has also been used to create and test interventions (for an overview see Holmes & Bourne, 2008; James et al., 2016).

Dibbets and Schulte-Ostermann (2015) signalled a possible drawback of the TFP; watching films is a somewhat passive endeavour that lacks active behavioural engagement. The participant remains an outsider to the film scenes. Being able to immerse in the film’s environment should increase the participant’s ‘feeling of presence’, which is commonly described as ‘the feeling of being there’, even though you ‘know’ you are not (Wirth et al., 2007). Virtual reality (VR) may provide a good alternative, because it can induce a greater feeling of presence than watching a film on a two-dimensional screen, which may lead to more realistic (Slater, 2009) and more emotional (Riva et al., 2007) responses to portrayed events. Noteworthy in this respect is also a different line of research, in which idiosyncratic autobiographical memories of healthy participants are often used to test the effects of dual-task interventions (van den Hout & Engelhard, 2012). An obvious disadvantage of this approach is that the age of the traumatic events underlying such memories differs between participants. This is problematic because older and stronger memories are less likely to be modified after reactivation than younger and weaker ones (Schwabe, Nader, & Pruessner, 2014). Like the TFP, the VR paradigm solves the problem of experimental control. However, like ‘real-life’ autobiographical events, VR allows participants to be the ‘protagonist’ and aversive events to be experienced ‘directly’. Moreover, the ability to interact may further increase the feeling of presence (Sanchez-Vives & Slater, 2005), and it provides new opportunities to investigate a range of PTSD-predicting factors that cannot be investigated using a ‘static’ film (e.g. sense of control over the traumatic event).

Recently, Dibbets and Schulte-Ostermann (2015) published the first study aimed at developing a fitting VR analogue to real-life trauma, by comparing the TFP with a VR scene with respect to changes in negative mood and the development of intrusive memories. The VR scene resulted in more immersion but did not result in stronger changes in negative mood or more intrusions. In fact, intrusion distress was higher after watching the film than after VR. The authors proposed that the VR scene may have been less intense than the film. Cuperus, Laken, van den Hout, and Engelhard (2016) argued that another explanation may be the lack of interactive features of the VR scene, which were limited to the ability to determine one’s distance to the event as a passive observer of the scene. They explored the utility of a VR paradigm with more interactive features, in which participants had to navigate through a virtual manor that was designed to induce fear. The aversive events in this environment were directed at the participants themselves and were triggered by their actions and decisions. Some of these events (e.g. a poltergeist spawning nearby) are ‘implausible’, but the VR scene induced vivid and unpleasant memories, which suggests that it may be a useful method of inducing negative memories. In the present study, we aimed to further validate its use as an experimental model to study psychological trauma by comparing its effects on intrusive memory development and mood with those of the well-established TFP. Vividness and emotionality ratings of recalled memories related to the film or VR scene were also compared, and participants filled out an evaluation questionnaire which contained statements about the film or VR scene (e.g. *I felt personally involved*).

Bayesian analysis was used to evaluate our hypotheses (Hoijtink, 2012; Mulder, Hoijtink, & De Leeuw, 2012). Although the aversive events in the VR scene are likely to be considered scary and/or threatening, they are much less aversive than those of the film we used, which largely consists of acts of rape and physical violence. VR is probably superior in terms of feeling of presence, which may compensate for the difference in content. The first hypothesis was therefore that the VR scene would elicit an equal amount of intrusions as the film. James et al. (2016) advised researchers to use a film that is sufficiently aversive to model trauma. Therefore, from an ethical point of view, it may be advisable to use the VR scene instead of highly aversive film material if both methods are equally effective in terms of intrusion frequency. Nevertheless, given that many studies have found that TFP is effective (Holmes & Bourne, 2008; James et al., 2016) and that the VR paradigm is relatively novel, the second hypothesis was that the film would elicit more intrusive memories. Finally, because the qualities of VR may overcompensate the less aversive content, we also tested the contrasting third hypothesis that the VR scene would elicit more intrusive memories. Following the same rationale, we tested the same three hypotheses with respect to vividness and emotionality of the negative memories induced by both paradigms. We expected pre- to post-film/VR mood changes and the ratings of the four statements of the evaluation questionnaire to follow the pattern to be observed for intrusion frequency.

## 1. Method

### 1.1. Participants

Participants were recruited via the website ‘proefbunny.nl’, and a Facebook recruitment page for experiments at Utrecht University (‘Universiteit Utrecht Betaalde Experimenten’). To be eligible, participants had to be at least 18 years old. Individuals with a medical history of heart disease or epilepsy, or with psychiatric problems, were excluded. Fifty participants were assigned randomly, but with gender ratio controlled for, to the film condition (nine males, 16 females) or the VR condition (eight males, 17 females). Most of them were students at Utrecht University. They participated in exchange for remuneration or course credits. Their mean age was 22.2 years (range 19–31; *SD* = 3.0); 22.6 years in the film condition (*SD* = 3.5), and 21.7 years (*SD* = 2.4) in the VR condition.

### 1.2. Ethical considerations

The study was approved by the Ethical Committee of the Faculty of Social and Behavioral Sciences of Utrecht University (FETC16-013). We adopted the safety strategies from the study of Cuperus et al. (2016). In the present study, however, participants were not informed about the nature of the VR scene and the film in the acquisition text, because we did not want to exclusively attract fans of the horror genre. Instead, prior to the day of the experiment, they were sent the information letter which contained this information, so they still had time to think about participation.

### 1.3. Measures

#### 1.3.1. Neuroticism

The 22 items from the neuroticism scale of the Eysenck Personality Questionnaire (EPQ; Eysenck & Eysenck, 1975) were used to assess neuroticism. Items were rated ‘yes’ or ‘no’, which translates to scores of 1 or 0, or vice versa, depending on the question. Higher scores indicate greater neuroticism. For the present study Cronbach’s α was .78.

#### 1.3.2. State and trait anxiety

The State-Trait Anxiety Inventory (STAI; Spielberger, Gorsuch, & Lushene, 1970) was used to assess anxiety. The test is split into the S-Anxiety scale and the T-Anxiety scale, measuring state anxiety (Cronbach’s α = .89) and trait anxiety (Cronbach’s α = .88) respectively, and each having 20 items. Items of both scales were rated on 4-point scales that ranged from 1 (not at all/almost never) to 4 (extremely/almost always). Higher scores indicate greater anxiety.

#### 1.3.3. Mood

Ratings of mood (happy, anxious, depressed, and angry) were given before and after the film or VR scene, on four 100 mm visual analogue scales (VAS) that ranged from 0 (not X at all) to 100 (extremely X; cf. Davies & Clark, 1998).^1^

#### 1.3.4. Memory vividness and emotionality

Participants were asked to recall the moment from the film or VR scene that they considered most unpleasant. They were instructed to visualize this moment and keep an image of it in mind for 10 s, and then rate its vividness and emotionality on two 100 mm VAS that ranged from 0 (not vivid/unpleasant) at all to 100 (extremely vivid/unpleasant; cf. Engelhard, van den Hout, & Smeets, 2011).^2^

#### 1.3.5. Evaluation questionnaire

This questionnaire contained four statements about the film or VR scene: (1) *I felt personally involved*, (2) *The events were unpredictable*, (3) *What happened somehow seemed real*, and (4) *I was startled by what happened*. Participants rated these statements on four 100 mm VAS that ranged from 0 (not X at all) to 100 (extremely X).

#### 1.3.6. Intrusions

Intrusive memories were recorded in a tabular paper-and-pencil intrusion diary for seven days after watching the film/VR (Holmes et al., 2004). Participants noted each intrusion’s content and rated whether it was an image, a thought, or a combination of both. For the present study, intrusions are defined as unintended, spontaneously occurring memories that at least contain an image, so mere thoughts were excluded.

#### 1.3.7. Diary compliance

Participants rated the statement *I was often unable (or often forgot) to report my intrusions in the diary* on an 11-point scale that ranged from 0 (totally untrue) to 10 (totally true).

### 1.4. Procedure

After reading the information sheet, participants signed the consent form. They were then shown a neutral film, which was a 1:51 min scene from the movie ‘Coach Carter’ (Gale, Robbins, Tollin, & Carter, 2005), after which they filled out the EPQ and the STAI, and rated their mood. Depending on random assignment, participants were then shown the trauma film or VR scene.

### 1.5. Film condition

The trauma film consisted of four scenes depicting acts of violence and rape from the movie ‘Irréversible’ (Chioua, Cassel, & Noé, 2002), lasting 6:50 min in total (1 × 140 s, 3 × 90 s; cf. Henckens, Hermans, Pu, Joëls, & Fernández, 2009). Clips from this movie induced intrusive memories in several studies (e.g. Schaich, Watkins, & Ehring, 2013; Verwoerd, De Jong, & Wessel, 2008). Furthermore, a variety of physiological measures (cortisol level, heart rate, and pupil dilation) confirmed successful stress induction for these particular four scenes (Henckens et al., 2009), and a longer version of the rape scene elicited a higher heart rate, more distress, and more intrusive memories than three other trauma films (Weidmann, Conradi, Gröger, Fehm, & Fydrich, 2009). Participants were instructed to immerse completely into the depicted film scenes, after which the experimenter turned off the light and left the room (cf. Dibbets & Schulte-Ostermann, 2015). The film scenes were projected on a 16.93 × 11.49-inch screen and audio was provided through a headphone (Sennheiser HD 449). Participants started the film by pressing the space bar and were asked to notify the experimenter when it was finished.

### 1.6. VR condition

The VR scene was a modified version of ‘Affected’ version 1.55, developed by Fallen Planet Studios (fallenplanetstudios.com), made to fit the needs of the present study. Unlike in the original version as used by Cuperus et al. (2016), in this modified version participants started in the ‘Manor’ stage instead of another room where the stage had to be selected first. Furthermore, it contained no random events and only allowed for one route in the manor, so we could be sure that all participants were exposed to the same events.

The environment of the manor is generally scary and contains several jump scares, such as a slamming door, a cabinet falling over, and a poltergeist that spawns nearby. The goal was to reach the other end of the manor by crossing each section and jump scare once. Participants were instructed to notify the experimenter when the end was reached. They were also informed that the experimenter would leave the room after the VR scene was started. To prevent that duration of exposure to VR would exceed the length of the trauma film, the experimenter re-entered the room and turned it off after 6:50 min (film duration) if it was not yet finished by then.

Participants moved through the virtual environment using a Microsoft Xbox 360 controller. The visuals were provided through a head-mounted display (Oculus Rift Development Kit 2), and audio was provided through a headphone (Sennheiser SD 449).

After the film or VR scene, participants rated the mood scale again. We then used a distractor task to remove film- or VR-related visuals from working memory. It was a paper-and-pencil Sudoku puzzle, taken from an online database and ranked level ‘easy’ (cf. Tadmor, McNally, & Engelhard, 2016). Participants were asked to complete as much of the puzzle as possible within 90 s. Afterwards, they recalled the moment from the film or VR scene that they considered most unpleasant and rated the vividness and emotionality of this memory. Finally, they filled out the evaluation questionnaire and were given the intrusion diary. The experimenter guided them through the written instructions that were included with the diary to make sure that these were clear to them.

Participants returned to the laboratory one week later to hand over the diary and discuss the reported intrusions with the experimenter. They also rated diary compliance, after which they were debriefed and offered a short mindfulness session of approximately 5 min.

### 1.7. Data analyses

Before analysing the data, an analysis plan was formulated. Because neuroticism is related to PTSD symptoms (e.g. Engelhard, van den Hout, & Lommen, 2009; van den Hout & Engelhard, 2004), it was included as a covariate in the analyses. As a result, the hypotheses concern the conditional means. The anxiety variables were added as descriptive statistics.

We formulated our expectations regarding the three key variables, intrusion frequency, memory vividness, and memory emotionality, in hypotheses:

H1: µ_film_ = µ_VR_

H2: µ_film_ > µ_VR_

H3: µ_film_ < µ_VR_

The first hypothesis states that the two conditions have equal means on the variable of interest. The second hypothesis specifies that the mean of the relevant variable in the film condition is higher than the mean in the VR condition. The third hypothesis states that the mean in the film condition is lower than in the VR condition. Together, H1, H2, and H3 form all possibilities of equality and inequality between the two means.

A frequentist analysis cannot quantify the relative evidence for a set of null (H1) and inequality constrained (H2 and H3) hypotheses (Wagenmakers, 2007). This is possible using Bayes factors and posterior probabilities. The Bayes factor BF_12_ expresses the support for H1 relative to H2. For example, if BF_12_ = 1, both hypotheses are equally supported by the data, if BF_12_ = 3, H1 is three times more supported by the data than H2, and if BF_12_ = .25, H2 is four times more supported than H1. Some guidelines for interpretation have been proposed by Kass and Raftery (1995), suggesting that a Bayes factor of 3 (or .33) indicates ‘substantial evidence’, and a Bayes factor of 10 (or .10) indicates ‘strong evidence’. However, we like to emphasize that these are merely guidelines and that, for instance, Bayes factors of 2.8 or 3.1 express rather similar evidence.

The Bayes factors can be used to update prior probabilities of the hypotheses into posterior probabilities that can be used to easily evaluate the relative support for more than two hypotheses given the observed data (Hoijtink, 2012, p. 53). In the present study, we assumed that a priori each of the hypotheses is equally likely, that is, the prior probabilities are equal for each hypothesis considered.

Bayes factors were computed in BIEMS (Mulder et al., 2012). In order to compute a Bayes factor, a prior distribution for the parameters of the statistical model needs to be specified under each hypothesis. BIEMS computes a suitable (conjugate) prior distribution using a minimal training sample from the data (Mulder et al., 2012). Thus, the prior distribution is based on the data and does not incorporate additional prior information. This results in a so-called default Bayes factor.

Eight variables (the mood and questionnaire variables) were selected for further exploratory analyses. In these analyses, the same set of hypotheses was considered as in the analyses of the key variables (H1, H2, and H3).

For intrusion frequency, one score deviated more than three standard deviations from the mean. Before analysing the data, this score was changed to one unit larger than the next most extreme score in the distribution (Tabachnick & Fidell, 1996).

## 2. Results

### 2.1. Baseline variables

Table 1 presents the means and standard deviations for the baseline variables, key variables, and exploratory variables. Both state and trait anxiety were comparable between the two randomised groups. However, we found that despite random assignment the VR group, on average, scored higher on neuroticism.

**Table 1.** Mean scores (*SD*) for the baseline variables, key variables, and exploratory variables.

Variable	Film	VR
Baseline variables		
Neuroticism	4.56 (3.07)	6.80 (4.37)
State anxiety	34.68 (8.29)	35.24 (7.33)
Trait anxiety	33.12 (8.94)	32.44 (5.41)
Key variables		
Intrusion frequency	3.60 (3.83)	3.48 (3.97)
Memory vividness	67.60 (18.20)	62.72 (22.93)
Memory emotionality	69.48 (21.55)	51.08 (25.40)
Exploratory variables		
Happy (post – pre)	−13.92 (18.38)	−17.48 (12.07)
Anxious (post – pre)	11.24 (19.65)	28.80 (21.01)
Depressed (post – pre)	12.36 (16.91)	11.20 (17.84)
Angry (post – pre)	17.12 (19.46)	2.48 (12.37)
Personal involvement	45.84 (31.39)	63.52 (18.87)
Unpredictability	38.40 (23.18)	55.08 (19.24)
Realism	55.52 (26.91)	60.52 (19.06)
Startle	63.08 (24.62)	72.00 (20.12)

### 2.2. Main analyses

Table 2 depicts the Bayes factors and posterior probabilities for H1, H2, and H3 for each of the key variables. The preferred hypothesis differs per variable of interest. With respect to intrusion frequency, the best hypothesis is H1, which states that the VR scene would elicit an equal amount of intrusions as the film. With respect to memory vividness it seems that, although H1 has more than half of the posterior probability, H2 also has substantial probability and cannot be easily ruled out. Thus, either memory vividness was equal for both conditions or it was higher for the film condition. However, for memory emotionality the best hypothesis is H2, which states that memory emotionality would be higher in the film condition than in the VR condition.^3^

**Table 2.** Bayes factors and posterior probabilities for key variables; H1, H2, and H3 (controlling for neuroticism; EPQ).

Variable	BF_12_	BF_13_	BF_23_	PP H1	PP H2	PP H3
Intrusion frequency	3.58	5.32	1.48	.681	.190	.128
Memory vividness	1.63	8.36	5.13	.577	.354	.069
Memory emotionality	.06	6.22	102.33	.056	.921	.009

### 2.3. Exploratory analyses

Table 3 presents the Bayes factors and posterior probabilities for H1, H2, and H3 for all exploratory variables. It shows that there is no clear trend over the variables. For the mood variables, it seems most likely that participants in the film condition had a larger increase in anger than in the VR condition (H2 was supported the most). The increase in anxiety was most likely larger in the VR condition than in the film condition (H3 was supported the most). For the variables ‘happy’ and ‘depressed’ it appears that we cannot easily choose the best hypothesis.

**Table 3.** Bayes factors and posterior probabilities for exploratory variables; H1, H2, and H3 (controlling for neuroticism; EPQ).

Variable	BF_12_	BF_13_	BF_23_	PP H1	PP H2	PP H3
Happy^a^	1.14	3.72	3.26	.467	.408	.125
Anxious	10.00	.05	<.01	.048	.005	.948
Depressed	2.18	2.46	1.13	.536	.246	.218
Angry	.04	8.00	199.00	.038	.957	.005
Personal involvement	6.57	.24	.04	.186	.028	.785
Unpredictability	15.00	.08	.01	.070	.005	.925
Realism	3.35	1.53	.46	.512	.153	.335
Startle	5.00	.59	.12	.347	.069	.584

^a^Higher scores indicate less reduction of happiness ratings.

In the second set of exploratory variables, we found that for ‘personal involvement’ and ‘unpredictability’, both H1 and H2 are unlikely hypotheses relative to H3, indicating that it is most likely that the VR scene was more personally involving and unpredictable than the film. Additionally, for the variables ‘realism’ and ‘startle’, H1 and H3 have more weight than H2, but it is not clear which hypothesis is most supported. Thus, it is inconclusive whether the film and VR scene were equally realistic and startling (H1) or whether VR was more realistic and startling (H3).^4^

## 3. Discussion

The TFP is a well-established method to study the effects of analogue psychological trauma under controlled laboratory settings (for an overview see Holmes & Bourne, 2008; James et al., 2016). However, because watching films is a somewhat passive endeavour that lacks active behavioural engagement, VR may provide a better alternative (Dibbets & Schulte-Ostermann, 2015). In the present study, we aimed to further validate the VR paradigm used by Cuperus et al. (2016) as an experimental model to study psychological trauma by comparing its effectiveness with the TFP.

The results indicate that the film and VR scene were equally effective in inducing vivid and intrusive memories. This is noteworthy because we used a highly aversive film (‘Irréversible’), depicting physical and sexual violence, and a game-like VR scene (‘Affected’; Cuperus et al., 2016). As argued by James et al. (2016), in selecting a film, it is not necessarily the aim to find the most aversive film that an ethical committee will allow. They advised researchers to aim to find a film that is sufficiently aversive to model trauma. From an ethical point of view, it may be advisable to use the VR scene instead of the clips from ‘Irréversible’ to generate intrusive and vivid memories. This way, participants do not have to be exposed to highly aversive film material. However, watching the film did result in memories of higher emotional valence. In light of ethical considerations and the presumably beneficial qualities of VR (e.g. inducing a greater feeling of presence and allowing interaction with the environment), using the VR scene could be preferable and at least is worth further exploration.

With respect to the exploratory variables, the results were mixed. Participants in the film condition seemed to show a greater increase in anger than participants in the VR condition, which is likely the result of the morally objectionable content in the film. However, it appears that participants in the VR condition showed a greater increase in anxiety, which may be caused by the fact that the VR scene was specifically designed to induce fear. Another possibility may be that the film elicited a wider variety in emotional responses; anxiety and anger, but also horror and disgust (Hagenaars, Brewin, Van Minnen, Holmes, & Hoogduin, 2010), whereas the VR scene was specifically adequate in eliciting anxiety.

The results also indicated that the VR scene was considered more personally involving and unpredictable. Speculatively, the VR scene was considered more involving because it contained events that were directed at participants themselves. The higher unpredictability ratings in the VR condition may be caused by jump scares that are designed to be unpredictable. Note that intrusion frequency for VR and film seemed similar despite these differences, which may suggest that personal involvement and unpredictability are not relevant for intrusion development. However, theoretical models assume otherwise (e.g. Foa, Zinbarg, & Rothbaum, 1992). The influence of these factors is therefore more likely outweighed by the highly aversive content of the film compared to the VR content. For the remaining exploratory variables, the data appears insufficient to express a preference for a certain hypothesis.

One could argue that it is worth exploring more complex and/or aversive VR scenes although, from an ethical point of view, we can consider it a strength of our VR scene that it was not extremely aversive; that is, it may be aversive enough to study the development of intrusive, vivid, unpleasant memories. A direction for future research is to replicate this study with groups that have similar neuroticism scores. It would also be interesting to compare the VR scene with a two-dimensional version of the same scene. This would provide some insight into the link between feeling of presence and PTSD symptoms, and into how far the presumed greater feeling of presence is accountable for the results of the present study, as opposed to the difference in content. Alternatively, a feeling of presence measure such as the ITC-Sense of Presence Inventory (Lessiter, Freeman, Keogh, & Davidoff, 2001) could be integrated in the design of the present study.

## Supplementary Material

Chinese and Spanish abstract

## Notes

1. We intended to measure the effect of the film/VR scene on state dissociation as well, using the Dissociative State Subscale. However, we only included a post-test and decided not to report the data because of this methodological flaw.

2. For purely exploratory purposes, we also measured heart rate during another 1-min version of this recall task. Also, a 1-min baseline heart rate measurement was established before the film/VR scene, so that the effect of recall on heart rate could be derived from difference scores. However, due to interpretation difficulties we decided not to report these data.

3. The analyses were also executed without neuroticism as a covariate, because the small sample can cause bias in the conditional means. The results were similar, with the exception that none of the hypotheses were clearly preferred for intrusion frequency.

4. The exploratory analyses were also executed without neuroticism as a covariate, because the small sample can cause bias in the conditional means. The results were similar.

## Highlights of the article

- Healthy participants watched an aversive film or moved through a VR scene
- Main outcome measures were intrusion frequency over a 7-day period, and self-rated vividness and emotionality of recalled memories related to the film or VR scene
- Film and VR were equally effective in inducing intrusive memories
- Memories of the film and VR scene were equally vivid, but film-related memories were more emotional
- The VR scene merits further exploration considering ethical considerations and additional possibilities
