# Supplementary material for: A virtual reality paradigm as an analogue to real-life trauma: its effectiveness compared with the trauma film paradigm
Source: Eur J Psychotraumatol. 2017 Jun 14;8(sup1):1338106. doi: 10.1080/20008198.2017.1338106 (PMC6516735; doi:10.1080/20008198.2017.1338106)
Supplement: Chinese and Spanish abstract [file ZEPT_A_1338106_SM3092.zip › Chinese abstract.pdf]

用虚拟现实范式模拟真实生活创伤：和创伤电影范式的效果对比

Anne A. Cuperus, Fayette Klaassen, Muriel A. Hagenaars, Iris M. Engelhard

摘要

背景：创伤电影范式（Trauma Film Paradigm, TFP）是在有控制的实验室设置中研究类心理创伤的影响的一种成熟方法。它被用来检验创伤前、围创伤期和创伤后的加工过程，以及用以干预治疗和检验治疗效果。一个可能的弊端是，观看影片是一种多少有些被动的方式，缺乏主动的行为卷入。虚拟现实（Virtual Reality, VR）可能是一个更好的替代选择。和TFP一样，VR允许实验控制，同时引发更强烈的"存在感"，允许和环境进行互动，从而可以对行为-反应联结进行研究。

目的：本研究的目的是对VR范式和TFP进行效果对比，进而验证VR范式作为研究心理创伤的实验模型的可用性。

方法：一组参与者（N=25）观看了一部厌恶性影片，另一组参与者（N=25）观看了一段VR场景。主要的结果测量中使用了七天日记法测量闯入记忆的频率，并对和影片或者VR场景相关的回忆的逼真度和情绪强度进行自评。

结果：总的看来，结果显示影片和VR场景能同等有效地引发逼真的闯入记忆。然而，自评情绪强度结果显示，和影片相关的记忆所引起的情绪强于VR场景。

结论：观看影片比VR场景能更有效地引发情绪性的记忆，可能是因为影片的内容更加具有厌恶性。不过，VR场景在引发逼真的闯入记忆时具有同等效果，并值得进一步探索其符合伦理考虑（较少厌恶性内容）和其它可能的优点（比如引发更强的感和允许与环境互动）。

关键词：创伤后应激障碍，闯入记忆，创伤电影范式，虚拟现实

(Abstract translated by Yulan Qing.)
