# Supplementary material for: A virtual reality paradigm as an analogue to real-life trauma: its effectiveness compared with the trauma film paradigm
Source: Eur J Psychotraumatol. 2017 Jun 14;8(sup1):1338106. doi: 10.1080/20008198.2017.1338106 (PMC6516735; doi:10.1080/20008198.2017.1338106)
Supplement: Chinese and Spanish abstract [file ZEPT_A_1338106_SM3092.zip › Spanish abstract.pdf]

El paradigma de realidad virtual como analogía del trauma de la vida real: su eficacia comparada con el paradigma de la película de trauma

Antecedentes: El paradigma de la película de trauma (PPT) es un método bien establecido para estudiar los efectos del trauma psicológico analógico en entornos controlados de laboratorio. Se ha utilizado para examinar procesos pre, peri- y post-traumáticos, y para crear y probar intervenciones. Un posible inconveniente es que ver películas es una tarea algo pasiva que carece de implicación conductual activa. La realidad virtual (RV) puede ofrecer una alternativa mejor. Al igual que el PPT, la RV permite el control experimental. Además, puede inducir una mayor "sensación de presencia" y permite la interacción con el entorno, lo que posibilita el investigar las asociaciones acción-reacción.

Objetivo: Pretendemos validar la utilidad del paradigma de RV como modelo experimental para el estudio del trauma psicológico comparando su eficacia con el PPT.

Método: A un grupo de participantes (N = 25) se les mostró una película aversiva y a otro grupo (N = 25) se le pasó una escena de RV. Las principales medidas de los resultados fueron la frecuencia de las intrusiones evaluadas con un diario de 7 días y una auto-evaluación de la viveza y la emotividad de los recuerdos evocados relacionados con la película o escena de RV.

Resultados: En conjunto, los resultados sugieren que la película y la escena de RV fueron igualmente eficaces para inducir recuerdos vívidos e intrusivos. Sin embargo, la intensidad emocional auto-reportada parecía ser mayor para los recuerdos relacionados con la película que para los recuerdos relacionados con la escena de RV.

Conclusiones: La película fue más eficaz que la escena de RV a la hora de inducir recuerdos emocionales, tal vez debido a su contenido más aversivo. Sin embargo, la escena de RV indujo recuerdos vívidos e intrusivos con la misma eficacia y merece una exploración adicional en base a consideraciones éticas (contenido menos aversivo) y a otras cualidades supuestamente beneficiosas (p.ej., inducir una mayor sensación de presencia y permitir la interacción con el entorno).
